# Hypertension with primary aldosteronism is associated with increased carotid intima‐media thickness and endothelial dysfunction

**DOI:** 10.1111/jch.13585

**Published:** 2019-06-12

**Authors:** Ahmet Demirkiran, Henk Everaars, Ali Elitok, Peter M. van de Ven, Yvo M. Smulders, Koen M. Dreijerink, Refik Tanakol, Mustafa Ozcan

**Affiliations:** ^1^ Department of Cardiology, Istanbul Faculty of Medicine Istanbul University Istanbul Turkey; ^2^ Department of Cardiology Amsterdam University Medical Center ‐ Vrije Universiteit Amsterdam Amsterdam the Netherlands; ^3^ Department of Epidemiology and Biostatistics Amsterdam University Medical Center ‐ Vrije Universiteit Amsterdam Amsterdam the Netherlands; ^4^ Division of Vascular Medicine, Department of Internal Medicine Amsterdam University Medical Center ‐ Vrije Universiteit Amsterdam Amsterdam the Netherlands; ^5^ Division of Endocrinology, Department of Internal Medicine Amsterdam University Medical Center ‐ Vrije Universiteit Amsterdam Amsterdam the Netherlands; ^6^ Division of Endocrinology, Department of Internal Medicine, Istanbul Faculty of Medicine Istanbul University Istanbul Turkey

**Keywords:** aldosterone, carotid intima‐media thickness, endothelial dysfunction, flow‐mediated dilation, hypertension, primary aldosteronism

## Abstract

Patients with primary aldosteronism induced hypertension are more likely to experience cardiovascular events compared to patients with essential hypertension. Primary aldosteronism may therefore have distinct adverse effects on cardiovascular structure and function, independent of hypertension. However, current data on such effects of primary aldosteronism are conflicting. The aim of the present study was to investigate the influence of primary aldosteronism on vascular structure and endothelial function, using intima‐media thickness as a vascular remodeling index and flow‐mediated dilation as a functional parameter. In total, 70 participants were recruited from patients with resistant hypertension. Twenty‐nine patients diagnosed with primary aldosteronism and 41 patients with essential hypertension were prospectively enrolled. Primary aldosteronism was due to aldosterone‐producing adenoma in 10 cases and due to idiopathic adrenal hyperplasia in 19 cases. All patients underwent ultrasound of the common carotid intima‐media thickness and flow‐mediated dilation of the brachial artery. Primary aldosteronism patients had significantly lower flow‐mediated dilation (3.3 [2.4‐7.4] % vs 14.7 [10.3‐19.9] %, *P* < 0.01) and significantly higher carotid intima‐media thickness (0.9 [0.7‐1.0] mm vs 0.8 [0.6‐0.9] mm, *P* = 0.02) compared to patients with essential hypertension. These differences remained significant after adjusting for age, sex, diabetes mellitus, 24‐hours systolic blood pressure, and smoking (*P* < 0.01). No differences in either outcome were observed between the adenoma and adrenal hyperplasia groups (both *P* > 0.05). Hypertensive patients with hyperaldosteronism appear to exhibit deteriorative effects on both vascular structure and function, independent of hypertension.

## 1 INTRODUCTION

Primary aldosteronism (PA) is defined as overproduction of aldosterone by the adrenal glands together with suppression of renin production. PA can result from either aldosterone‐producing adenoma (APA) or idiopathic adrenal hyperplasia (IAH) and has a high prevalence of approximately 11% among de novo hypertensive patients who are referred to a specialized center.^1^ Recent studies indicate that patients with PA are more likely to experience cardiovascular events and renal complications compared to patients with essential hypertension (EH).^2^, ^3^, ^4^ These findings suggest that primary aldosteronism might have distinct adverse effects on cardiovascular structure and function, independent of hypertension.

Aldosterone overproduction leads to changes in vascular morphology, such as smooth muscle hypertrophy and collagen deposition within the extracellular matrix.^5^, ^6^ As a result, patients with PA have more pronounced fibrosis in the small resistance arteries.^7^ Intima‐media thickness (IMT), which can be assessed through ultrasound of the common carotid artery, is used as a marker of atherosclerosis.^8^ Early studies reported that common carotid intima‐media thickness (CC‐IMT) is higher in patients with PA compared to patients with EH patients.^9^, ^10^ However, these studies were performed more than a decade ago. A more recent study reported no significant differences in baseline CC‐IMT between subtypes of PA and EH patients.^11^ Therefore, it remains debatable whether the subtypes of PA (ie, APA and IAH) exhibit different effects on cardiovascular structure.

Besides vascular structure, aldosterone also has profound effects on endothelial function. Aldosterone reduces endothelium‐dependent vasodilation, either directly or through angiotensin II. In experimental studies, it has been demonstrated that aldosterone prevents inducible nitric oxide (NO) synthase.^12^ Flow‐mediated dilation (FMD), which can be assessed with ultrasound of the brachial artery pre‐ and post‐occlusion, is a widely used tool to directly and non‐invasively evaluate endothelium‐dependent vascular function.^13^ Numerous studies have demonstrated that FMD is associated with the development of cardiovascular disease.^14^, ^15^ Although some studies reported that hypertensive patients with PA have lower FMD compared to patients with EH,^16^, ^17^ endothelial function paradoxically improves after 2 weeks of aldosterone excess.^18^ Recently, it has been suggested that the detrimental effects of aldosterone overproduction are much less prominent in IAH than in APA, causing patients with PA due to IAH to have similar endothelial function as patients with EH.^19^

Given the high prevalence (20%) of PA among patients with resistant hypertension, it is crucial to further elucidate the pro‐atherogenic role of PA.^20^ Therefore, the aim of the present study was to investigate the influence of PA and its subtypes on vascular structure and function, as assessed by CC‐IMT and FMD.

## 2 MATERIALS AND METHODS

Twenty‐nine patients diagnosed with PA were prospectively enrolled. PA was due to APA in 10 (34%) cases and due to IAH in 19 (66%) cases. Forty‐one patients with EH served as control group. Participants were consecutively recruited from patients with resistant hypertension referred to the endocrinology or cardiology department of the Istanbul University of Medicine between April 2012 and November 2015. Resistant hypertension was defined as blood pressure above >140/90mm Hg in spite of optimal doses of 3 antihypertensive drugs, including at least one diuretic, or blood pressure requiring ≥4 antihypertensive drugs to control.^21^ Patients with secondary causes of hypertension other than PA, such as Cushing’s syndrome and renovascular hypertension, were excluded from the study.

Both PA and EH patients were subjected to a similar antihypertensive treatment regimen. In order not to interfere with the renin‐angiotensin‐aldosterone system, pharmacological antihypertensive treatment was switched to monotherapy with a α‐blocker (doxazosin) or duo‐therapy with doxazosin and slow release verapamil. Oral potassium replacement was administrated to hypokalemic patients (<3.5 mEq/L) until a plasma level of >4.0 mmol/L was achieved. The first study procedures were performed at least 14 days after adjustment of therapy. Demographics, medical history and current medication were collected. In addition, office blood pressure was measured twice on the right upper arm after a 15‐minutes resting period using a manual sphygmomanometer. All patients also underwent 24‐hours ambulatory blood pressure monitoring (SpaceLabs 90207; Medical). The study was conducted in accordance with the Declaration of Helsinki, and the study protocol was approved by the institutional review board of the Istanbul University, Istanbul Faculty of Medicine. All patients provided written informed consent for the study.

### 2.1 Diagnosis of primary aldosteronism

Screening and diagnosis of PA were conducted according to the guidelines of the Endocrine Society.^22^ As a first step, screening for PA was performed by sampling plasma aldosterone concentration (PAC) and plasma renin activity (PRA). Patients with PAC > 15 ng/dL and plasma aldosterone/renin ratio (ARR) >20 were subjected to a saline infusion test or captopril challenge test to confirm the diagnosis of PA.

Saline infusion tests were regarded as positive if PAC was still >10 ng/dL following a 4‐hours, 2L intravenous infusion of saline. In 5 patients with ARR > 20 and severe systolic hypertension, saline infusion was replaced by a captopril challenge test. Captopril challenge tests were regarded positive if PAC remained elevated and PRA remained suppressed 1 hour after receiving 25‐50 mg of captopril orally.^22^ In patients with confirmed PA, subtype classification was performed using computed tomography (CT) and/or magnetic resonance (MR) imaging of the adrenal glands.

### 2.2 Echocardiography

All patients underwent standard echocardiography using two‐dimensional M‐mode and Doppler flow velocity as part of routine clinical care (GE Vivid 7.0, General Electric Vingmed Ultrasound) and according to the recommendations of the American Society of Echocardiography.^23^ Interventricular septum thickness (IVST) and posterior wall thickness (PWT) were measured at end‐diastole. In addition, pulse‐wave Doppler echocardiography was performed to determine E/A as a parameter of diastolic dysfunction.

### 2.3 Assessment of intima‐media thickness

Ultrasound of the carotid artery was performed using a multifrequency (12 MHz) linear array transducer (GE Vivid 7.0, General Electric Vingmed Ultrasound), according to the latest Mannheim carotid intima‐media thickness consensus statement.^24^ Patients with previous carotid angioplasty or carotid endarterectomy and patients with carotid plaques more than 2.0 mm were excluded. All measurements were performed on high‐resolution B‐mode images. For CC‐IMT measurements, patients were placed in the supine position with tilting of the head in a 45°C, opposite to the side of measurement. Longitudinal B‐mode images of the distal segment (10‐15 mm proximal to the carotid bifurcation) of the right common carotid artery were obtained in three consecutive regions of the common carotid artery at the far wall. Next, the distance between the blood‐intima and media‐adventitia interfaces was measured for all three regions. CC‐IMT was thereafter computed by averaging the values of measurements.

### 2.4 Assessment of flow‐mediated dilation

Endothelium‐dependent FMD was evaluated as described previously, using a multifrequency (12 MHz) linear array transducer.^25^, ^26^ Patients were placed in supine position, in a quiet, air‐conditioned room (22‐25°C), 15 minutes prior to the examination. High‐resolution B‐mode images of the brachial artery were obtained in the longitudinal plane, 3‐5 cm proximal to the elbow of the right arm. Care was taken to maintain probe position during the investigation. Reactive hyperemia was induced by inflating a blood pressure cuff around the forearm for 4‐5 minutes at 50 mm Hg above systolic blood pressure, followed by prompt deflation. Brachial artery diameter was assessed by measuring the distance between the two lumen‐intima interfaces at end‐diastole. Three measurements were performed at baseline and post‐occlusion. Baseline and post‐occlusive measurements were subsequently averaged. FMD was defined as the relative increase in brachial artery diameter ((diameter post‐occlusion − basal diameter)/basal diameter) × 100)).

Echocardiography, assessment of intima‐media thickness, and assessment of flow‐mediated dilation were performed by consensus of two experienced cardiac ultrasonographers (AD and AE), who were blinded to clinical data (eg, cause of hypertension).

### 2.5 Statistical analysis

Continuous variables are presented as mean ± standard deviation or median with interquartile range and were compared between two groups using the student *t* test for normally distributed data and Mann‐Whitney test for non‐parametric data. Categorical variables are expressed as frequency (percentage) and were compared between two groups using a chi‐square test. Multiple group comparisons were performed by one‐way analysis of variance (ANOVA), Kruskal‐Wallis, or chi‐square test according to variable type and distribution. Linear regression analysis was used to assess whether differences in FMD and CC‐IMT remained significant after correcting for age, sex, diabetes mellitus, 24‐hours systolic and diastolic blood pressure, and smoking. Association between continuous variables was quantified by Spearman’s correlation. Log transformation was applied to hormone levels in order to achieve parametric distribution. *P*‐values <0.05 were considered statistically significant. Statistical analysis was done with the Statistical Package for Social Sciences software (IBM SPSS statistics 22 for Windows).

## 3 RESULTS

Clinical characteristics of the study cohort are listed in Table 1. Patients with PA matched well with EH patients for age, gender, body mass index, and other cardiovascular risk factors. In addition, no differences in 24‐hours average systolic and diastolic blood pressures were present, nor did the duration of hypertension differ between the groups. This was also demonstrated by similar IVST, PWT, and diastolic dysfunction. Furthermore, there were no significant differences for chronic antihypertensive medication between patients with PA and EH. With regard to kidney function, patients with PA had a higher prevalence of proteinuria (41% vs 17%, *P* = 0.02) and slightly higher levels of creatinine (1.00 [0.6, 1.5] vs 0.8 [0.5, 1.4], *P* = 0.03) compared to patients with EH.

**Table 1:** Clinical characteristics

	Primary aldosteronism		Essential hypertension (n = 41)	*P*‐Value between groups
	APA (n = 10)	IAH (n = 19)		
Age (y)	45 ± 14	56 ± 13	52 ± 15	NS
Sex (male)	3 (30%)	11 (57%)	17 (41%)	NS
BMI (kg/m^2^)	25.4 ± 4.9	24.8 ± 2.3	24.1 ± 3.1	NS
IVSd (cm)	1.2 ± 0.2	1.2 ± 0.1	1.1 ± 0.1	NS
PWd (cm)	1.1 ± 0.2	1.1 ± 0.2	1.1 ± 0.1	NS
Diastolic dysfunction	4 (40%)	15 (78%)	22 (53%)	NS
Systolic BP (ABPM, mm Hg)	155 ± 27	147 ± 25	153 ± 19	NS
Diastolic BP (ABPM, mm Hg)	94 ± 19	86 ± 13	86 ± 10	NS
Duration of HT (years)	12 (4‐23)	5 (1‐18)	10 (5‐10)	NS
Plasma cholesterol (mg/dL)	181 ± 25	194 ± 43	198 ± 42	NS
LDL cholesterol (mg/dL)	107 ± 31	119 ± 36	125 ± 34	NS
HDL cholesterol (mg/dL)	50 ± 14	49 ± 20	49 ± 13	NS
Triyglycerides (mg/dL)	123 ± 56	146 ± 61	144 ± 93	NS
Lipid lowering medication	0 (0%)	2 (10%)	7 (17%)	NS
Hypokalemia (<3.5 mEq/L)	8 (80%)*/**	6 (31%)*	1 (2%)	<0.01
Presence of proteinuria	2 (20%)	10 (52%)*	7 (17%)	0.01
Creatinine (mg/dL)	0.8 (0.6‐1.2)	1.0 (0.9‐1.1)*	0.8 (0.7‐0.9)	0.03
Chronic antihypertensive therapy				
MRA	3 (30%)	3 (16%)	6 (15%)	NS
β blockers	8 (80%)	14 (74%)	30 (73%)	NS
α blockers	2 (60%)	7 (37%)	14 (35%)	NS
ACEI	8 (80%)	12(63%)	27 (66%)	NS
ARB	2 (20%)	6 (32%)	14 (34%)	NS
CCB	7 (70%)	8 (42%)	19 (46%)	NS
Diuretics	5 (50%)*	15 (79%)	34 (83%)	NS

Abbreviations: ABPM, ambulatory blood pressure measurement; ACEI, angiotensin‐converting‐enzyme inhibitor; ARB, angiotensin receptor blocker; BMI, body mass index; BP, blood pressure; CCB, calcium channel blocker; EF, ejection fraction; HT, hypertension; IVSd, interventricular septum thickness; MRA, mineralocorticoid receptor antagonists; PA, primary aldosteronism; PWd, posterior wall thickness.

* *P* < 0.05 vs EH.

** *P* < 0.05 vs IAH.

As expected, hypokalemia was more frequently present in patients with PA compared to patients with EH (48% vs 2.4%, *P* < 0.01). Within the group of PA, hypokalemia was more frequently observed in patients with APA than patients with IAH (80% vs 31%, *P* = 0.01).

### 3.1 Intima‐media thickness

**Figure 1:**
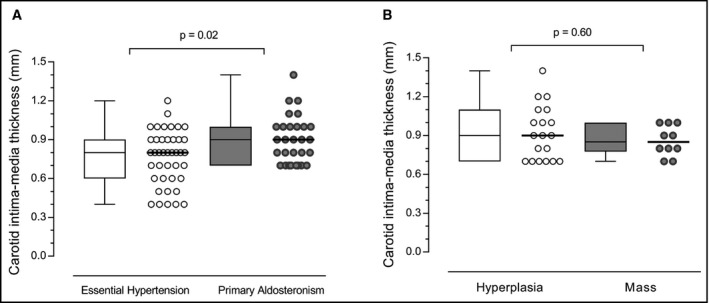
Intima‐media thickness in patients with PA and EH. Common carotid intima‐media thickness in patients with essential hypertension (EH) and primary aldosteronism (PA). Panel A displays the comparison between patients with EH and PA. Panel B displays the comparison between subtypes of PA. Data shown as median and interquartile range (Q1; Q3)

Figure 1A depicts CC‐IMT in patients with PA and EH. Patients with PA had significantly higher CC‐IMT compared to patients with EH (0.9 [0.7‐1.0] mm vs 0.8 [0.6‐0.9] mm, *P* = 0.02). This difference in CC‐IMT remained statistically significant after adjusting for age, sex, diabetes mellitus, 24‐hours systolic and diastolic blood pressure, and smoking (*P* < 0.01). Figure 1B displays comparison of CC‐IMT between the subtypes of PA. No differences in CC‐IMT were observed between patients with APA and IAH (0.9 [0.7‐1.0] mm vs 0.9 [0.7‐1.1] mm, *P* = 0.60).

### 3.2 Flow‐mediated dilation

**Figure 2:**
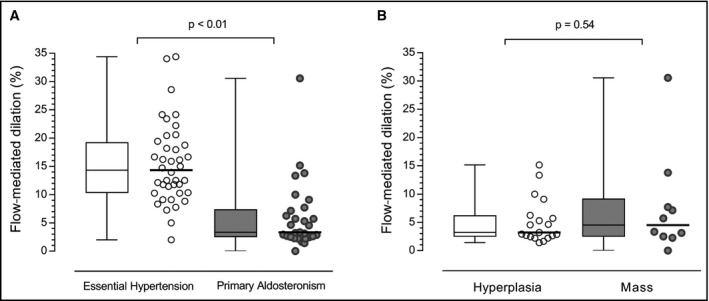
Flow‐mediated dilation in patients with PA and EH. Flow‐mediated dilation in patients with essential hypertension (EH) and primary aldosteronism (PA). Panel A displays the comparison between patients with EH and PA. Panel B displays the comparison between subtypes of PA. Data shown as median and interquartile range (Q1; Q3)

Figure 2A displays FMD in patients with PA vs EH. Patients with PA showed less FMD compared to patients with EH (3.3 [2.4‐7.4] % vs 14.7 [10.3‐19.9] %, *P* < 0.01). This difference in FMD also remained statistically significant after adjusting for age, sex, diabetes mellitus, 24‐hours systolic and diastolic blood pressure, and smoking (*P* < 0.01). Figure 2B illustrates the FMD between subtypes of PA. Patients with APA and IAH had similar FMD (4.5 [2.4‐9.2] % vs 3.2 [2.4‐6.2] %, *P* = 0.54).

### 3.3 Hormonal levels

As expected, patients with PA demonstrated higher PAC and ARR values and lower PRA values compared to patients with EH (*P* < 0.01). In addition, no significant difference was observed for PAC, ARR, and PRA between subtypes of PA (*P* > 0.05). Table 2 displays the hormonal values of patients with PA and patients with EH who had positive ARR (>20) and negative ARR during screening process of PA. Patients with PA showed higher PAC and ARR values and lower PRA values compared to patients with EH have negative ARR (*P* < 0.01), while there was no significant difference for hormonal values between patients with PA and patients with EH have positive ARR (*P* > 0.05).

**Table 2:** Hormonal data of the study population

	Primary aldosteronism (n = 29)	Essential hypertension (ARR [+]) (n = 18)	Essential hypertension (ARR [−]) (n = 23)	*P*‐Value between groups
PAC (ng/dL)	30.20 (18.0‐50.95)*	18.60 (10.82‐36.17)*	11.20 (6.24‐18.70)	<0.001
Plasma renin activity (ng/mL/h)	0.21 (0.08‐0.51)*	0.21 (0.08‐0.81)*	1.50 (0.82‐3.05)	<0.001
Aldosterone to renin ratio	103.35 (45.65‐308.0)*	60.70 (31.27‐133.25)*	7.10 (3.30‐10.40)	<0.001

Abbreviations: ARR, aldosterone/renin ratio; PAC, plasma aldosterone concentration.

* P < 0.05 vs EH (ARR [−]).

**Figure 3:**
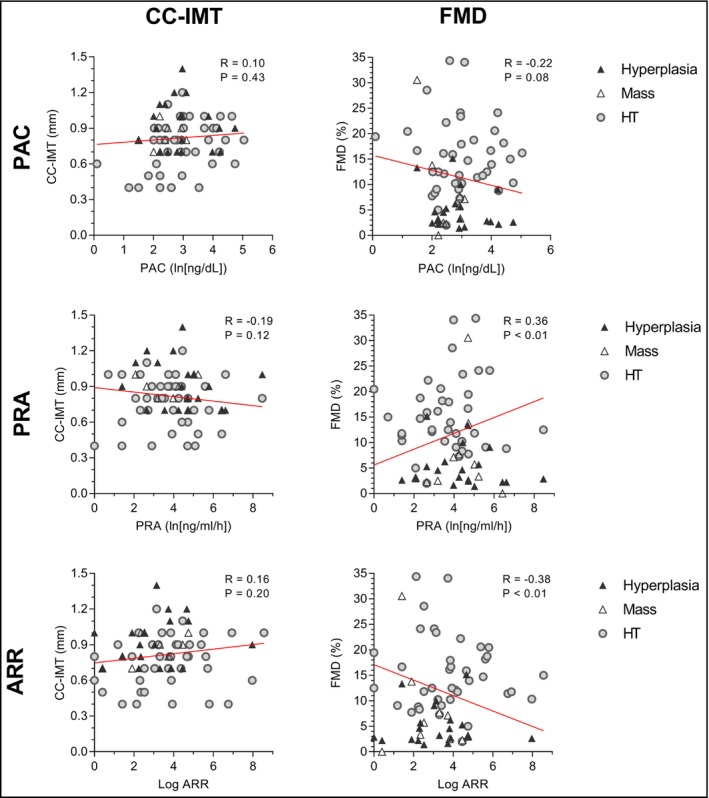
Relationship between FMD, CC‐IMT and hormone levels. Relationship between common carotid intima‐media thickness (CC‐IMT) (left), flow‐mediated dilation (FMD) (right), and levels of hormones pertaining to the renin‐angiotensin‐aldosterone system. Both patients with PA and EH are included (n = 70). EH and subgroups of PA are also represented with different symbols. ARR, aldosterone/renin ratio; EH, essential hypertension; PA, primary aldosteronism; PAC, plasma aldosterone concentration; PRA, plasma renin activity

Figure 3 demonstrates the relationship between IMT, FMD, and levels of hormones pertaining to the renin‐angiotensin‐aldosterone system, including both PA and EH patients. In the whole population, CC‐IMT did not correlate with levels of hormones (all *P* > 0.05). FMD, on the other hand, correlated significantly with ARR (*r* = −0.38, *P* < 0.01) and PRA (*r* = 0.36, *P* < 0.01), but not with PAC (*r* = −0.22, *P* = 0.08). After correcting for systolic blood pressure, both PRA and ARR were still significantly associated with FMD (both *P* < 0.05).

## 4 DISCUSSION

The present study is the first to perform combined assessment of vascular structure and function in patients with primary aldosteronism using flow‐mediated dilation and common carotid intima‐media thickness. The main findings are summarized as follows: (a) Patients with primary aldosteronism have inferior endothelial function and more arterial wall damage compared to patients with essential hypertension (b) Subtypes of PA may result in similar deleterious influences on vascular function and structure.

### 4.1 Primary aldosteronism as an independent risk factor for future cardiovascular events

Numerous retrospective and prospective studies indicated that patients with PA are at higher risk for cardiovascular events compared to patients with EH.^3^, ^4^, ^27^ Furthermore, it has been shown that patients with PA have higher incidences of proteinuria,^28^ cerebral hemorrhage,^29^ and left ventricular hypertrophy^30^ than age‐ and sex‐matched EH patients, regardless of blood pressure levels and duration of hypertension. Hence, it is highly likely that aldosterone itself and/or mechanisms related to overproduction of aldosterone have distinctive deteriorative influences on cardiovascular structure and function. Of note, increased aldosterone levels as a respond to salt deficiency are not associated with cardiovascular damage. Therefore, inappropriate aldosterone secretion for sodium levels seems the responsible mechanism for the increased cardiovascular risk in PA,^31^ although exact underlying pathology behind aldosterone‐related cardiovascular damage remains to be investigated. Recent evidence suggests that increased secretion of ouabain‐like compounds from adrenal cortex as a reaction to increased aldosterone results in raised blood pressure through vasoconstriction and thus may lead to cardiovascular damage, as well.^32^ Therefore, it is conceivable that level of both aldosterone and aldosterone‐related hormones can be a target in patients with PA to prevent cardiovascular events. In this regard, Hundemer at al. investigated potential of targeted medical approach to reduce cardiovascular risk in patients with PA.^33^ Patients with PA treated with mineralocorticoid receptor antagonists (MRA) demonstrated significantly higher incidences of cardiovascular events than patients with EH despite comparable blood pressure levels and cardiovascular risk profiles. Interestingly, the risk for incident cardiovascular events in patients with PA who were treated with more aggressive doses of MRA achieving a remarkable rise of renin was comparable with EH patients, whereas the excessed risk remained limited to the patients with PA whose renin activity remained suppressed suggesting tailored MR antagonist therapy may be a more effective approach to reduce the increased cardiovascular risk for patients with PA treated medically.

As a particular advantage of our study, we found no significant difference between patients with PA and EH for IVST, PWT, and diastolic dysfunction which supports a comparable blood pressure load. Consequently, our study suggests that PA affects both cardiovascular structure and function and does so independent of blood pressure. However, when evaluating parameters reflecting organ damage, only the percentage of proteinuria and levels of creatinine were higher in patients with PA compared to control. We hypothesize that differences in clinical and biochemical parameters are less prominent in our study due to relatively small number of the patients. Nevertheless, this study underlines the detrimental effect of hyperaldosteronism on vascular function and structure.

### 4.2 Subtype classification of primary aldosteronism

Therapeutic approaches in PA significantly and distinctly differ depending on the lateralization finding; thus, application of an accurate technique for subtype classification is great of importance. The recent guidelines of the Endocrine Society advocated AVS as the gold standard to distinguish between subtypes of PA suggesting use of CT as a stand‐alone technique only for young patients (<35 years) with spontaneous hypokalemia and marked aldosterone excess.^22^ However, CT may underdiagnose adenomas below 10 mm in diameter due to insufficient spatial resolution, whereas successive AVS demonstrates high sensitivity and specificity (95% and 100%, respectively) to distinct lateralization.^34^ In our study, although all PA confirmed patients underwent CT and/or MR imaging of the adrenals, subtype classification remains suboptimal due to unemployment of AVS. Of interest, AVS is not widely available worldwide due to challenging nature of the technique requiring high level of expertise. 35 Therefore, new alternative imaging methods are being developed such as NP‐59 scintigraphy scan and 11C‐metomidate PET‐CT, although none of them provide an adequate sensitivity and specificity for subtype differentiation of PA, yet.^36^

### 4.3 Vascular structure

Experimental studies have shown that excess of aldosterone stimulates vascular smooth cell hypertrophy, adventitial cell migration, and collagen accumulation in the extracellular matrix.^5^, ^6^, ^7^ These structural changes ultimately result in vascular remodeling which might explain the increased cardiovascular events in patients with PA. IMT is a valid and useful tool to investigate vascular structural changes. In several studies, a remarkable relationship was demonstrated between IMT and cardiovascular risk factors.^37^, ^38^ IMT is therefore accepted as a solid marker of the intermediate phase of atherosclerosis. In the present study, we found that patients with PA had significantly higher CC‐IMT compared to patients with EH. These findings are in line with previous studies. Two studies found that patients with PA have greater IMT compared to patients with EH.^9^, ^10^ Recent studies even demonstrated that IMT regressed in patients with PA who received specific medical and/or surgical treatment, suggesting that cardiovascular damage resulting from aldosterone overproduction is reversible.^11^, ^39^, ^40^

Although the relationship with PA and increased CC‐IMT is well established, data on the differences between subtypes are conflicting. While Matsuda et al reported that patients with IAH have higher CC‐IMT compared to patients with APA, Holaj et al observed no differences in CC‐IMT between subtypes.^11^, ^39^ However, in the study performed by Matsuda et al, patients with IAH were older and had higher blood pressure, which could explain the results. In the present study, we found no significant differences in CC‐IMT between subtypes of PA, which implies that influences of PA on cardiovascular structure in APA and IAH may not differ. However, differences in CC‐IMT may have been obscured due to the relatively small sample size. Therefore, larger studies comparing the effects of APA and IAH on cardiovascular structure are warranted.

### 4.4 Endothelial function

Aldosterone also exhibits adverse effects on endothelial function. Aldosterone was reported to directly limit NO production by decreasing NO synthase activity and increasing superoxide anion generation in endothelial cells.^41^, ^42^ Endothelium‐dependent vascular function can be directly assessed using FMD. Attenuated FMD is associated with several cardiovascular risk factors including hypertension, diabetes mellitus, dyslipidemia, and obesity.^43^ In addition, FMD was found to predict future cardiovascular events.^14^, ^15^ Several studies have investigated the influence of aldosterone on endothelial function. However, these studies have yielded conflicting results. Two studies reported lower FMD in PA compared with EH.^16^, ^17^ In addition, Hannemann et al investigated the relation between aldosterone levels and FMD in a general population of patients aged below 50 years. High and high‐normal aldosterone levels were associated with decreased FMD.^44^ Contrarily, Nietlispach et al observed an increase in endothelium‐dependent vasodilation after 2 weeks of pharmacologically induced aldosterone excess, which argues against a direct effect of aldosterone on endothelial function.^18^ In the present study, we detected significantly lower FMD in patients with PA compared to EH.

Recently, Matsumoto et al reported that APA is associated with inferior endothelial function, compared to patients with IAH.^19^ However, aldosterone levels were also found to be higher in patients with APA compared to patients with IAH. The attenuated post‐occlusive response can therefore not solely be attributed to subtype. In the present study, aldosterone levels were similar between patients with mass and hyperplasia and no differences in FMD were observed between the subtypes.

### 4.5 Hormonal data

In the present study, no correlation was found between IMT and hormone levels (PAC, PRA, ARR). These results are in accordance with literature.^9^, ^10^ Furthermore, a previous study found no correlation between PAC and regression of IMT after treatment of PA.^11^ These apparently inconsistent findings can be explained by several reasons. Firstly, blood samples were collected 2 weeks after discontinuation of medical therapy, and therefore, the hormonal levels during the chronic phase might differ. Secondly, additional factors such as density of aldosterone receptors may vary depending on the region and can be determinative in the occurrence and severity of the vascular damage, as well.

In the present study, FMD correlated significantly with ARR and also PRA, whereas no correlation was detected between FMD and PAC. The association of FMD with both ARR and PRA remained significant after correcting for blood pressure. Although some studies have reported that FMD is associated with hormone levels, this is still a matter of debate.^16^, ^17^, ^44^ Of note, ARR provides a more robust measure than PAC and PRA for evaluation of aldosterone overproduction, since PAC and PRA are affected by several confounders such as dietary sodium intake and posture.^45^ Consequently, ARR might be more closely associated with parameters of cardiovascular structure and function than PAC and PRA. However, CC‐IMT and FMD represent generalized atherosclerosis and other cardiovascular risk factors can still confound the relationship with ARR.

### 4.6 Implications and future perspectives

These data clearly confirm that exposure to inappropriate aldosterone overproduction for sodium levels is associated with increased cardiovascular risk by altering both cardiovascular structure and function. As a consequence, the pro‐atherogenic effects of PA need to be strictly monitored during daily clinical practice in patients with PA, even if blood pressure has normalized. Recently, the German Conn’s Registry showed that cardiovascular mortality is higher in patients with PA compared to patients with EH (50% vs 34%).^2^ Given the high prevalence (20%) of PA among patients with resistant hypertension,^20^ the relationship between subclinical atherosclerosis and PA and its subtypes requires further clarification in a large multi‐center trial with long‐term follow‐up. Periodic assessment of surrogate cardiovascular risk factors may help guide preventive strategies in patients with PA.

Finally, if hyperaldosteronism indeed exerts detrimental effects independent of blood pressure, surgical approach should be considered when possible to eliminate aldosterone excess. Furthermore, for medically treated patients with PA, dosing of medical therapy may be adjusted depending on aldosterone and renin levels to assess sufficient blockade of the adrenal mineralocorticoids.^33^ Further longitudinal studies demonstrating the impact of targeted application of MRA and surgical interventions on atherosclerotic biomarkers and cardiovascular outcome are warranted.

## 5 STUDY LIMITATIONS

This study has some important limitations. First, as previously mentioned, the number of patients with PA is relatively small, particularly for APA (10), which may have precluded the comparison between PA subtypes, as well as differences in clinical and biochemical parameters. Second, adrenal vein sampling (AVS), which is the preferred method for subtype classification of PA as recommended by the latest guideline of the Endocrine Society, was attempted in only four patients.^22^ Because of the low success rate of the procedure, the procedure was not performed in the remainder of patients. However, subtyping was performed using CT and/or MR imaging for all the patients with confirmed PA. Nevertheless, the results regarding comparison between subtypes of PA should be interpreted cautiously, since the distinction of subgroups is not definitive. Finally, a true control group is missing. However, the rationale of the study was able to assess the severity of atherosclerotic features in the patients with PA in comparison to patients with EH.

## 6 CONCLUSIONS

Primary aldosteronism appears to exhibit deteriorative effects on both vascular structure and function, as determined with carotid intima‐media thickness and flow‐mediated dilation, independent of hypertension.

## CONFLICT OF INTEREST

This study was initiated by the Istanbul University, Istanbul Faculty of Medicine, and the authors report no specific funding in relation to this research and no conflicts of interest to disclose.

## AUTHOR CONTRIBUTIONS

All authors have read and approved the submission of the manuscript. Prof. Ozcan, Prof. Tanakol, Assoc. Prof. Elitok, and Dr Demirkiran were involved in the conception and design of the study. Dr Demirkiran and Assoc. Prof. Elitok collected all study data. Dr Demirkiran and Dr Everaars were involved in the interpretation of data, as well as drafting the manuscript. Prof. Tanakol was involved in diagnosis and management of PA patients. Dr van de Ven performed the statistical analyses. Finally, Prof. Smulders and Dr Dreijerink aided in the interpretation of data, as well as in revising the manuscript for critically important intellectual content.
